# The influence of self-owned home blood pressure monitoring (HBPM) on primary care patients with hypertension: A qualitative study

**DOI:** 10.1186/1471-2296-12-143

**Published:** 2011-12-30

**Authors:** Adina Abdullah, Sajaratulnisah Othman

**Affiliations:** 1 Department of Primary Care Medicine, University of Malaya, Kuala Lumpur, Malaysia

## Abstract

**Background:**

Home blood pressure monitoring (HBPM) is gaining popularity among hypertensive patients. This study aimed to explore the influence of self-initiated HBPM on primary care patients with hypertension.

**Methods:**

Six in-depth interviews and two focus group discussions were conducted, taking into consideration the experiences of 24 primary care patients with hypertension. These patients had been using HBPM as part of their hypertension management. The overriding influences were grouped under themes which emerged from analyzing the data using the grounded theory approach.

**Results:**

There are both positive and negative influences of self-initiated HBPM. Patients used the readings of their HBPM to decide on many aspects of their hypertension management. The HBPM readings both influenced their adherence to diet and exercise and provided certain reassurance when they experienced symptoms. In addition, the act of discussing their HBPM readings with their health care providers resulted in an enhanced doctor-patient therapeutic relationship. Nevertheless, HBPM created confusion at times in some patients, particularly with regard to the target blood pressure level and the need for medication. This led to some patients making their own medical decisions based on their own standards.

**Conclusions:**

HBPM is becoming an integral part of hypertension management. Primary care patients who self-initiated HBPM reported being more self-efficacious, but lack of participation and guidance from their doctors created confusion, and hindered the true benefit of HBPM.

## Background

The use of HBPM as an adjunct to office blood pressure readings in the management of hypertension is advocated by the Malaysian National Hypertension Guideline [1] as well as many other international guidelines [2-6]. The advantages of home blood pressure readings include that they are more reflective of target organ damage [7,8], predictive of cardiovascular morbidity [9] and have the ability to detect the phenomena of both white coat and masked hypertension [10]. Better blood pressure control could be achieved in patients who monitor their blood pressure at home [11,12]. In a meta-analysis of 18 randomized control trials, patients using HBPM were found to have an improvement of approximately 2.2 mmHg systolic and 1.9 mmHg diastolic blood pressure. Although this reduction was modest, it is likely that this might contribute to an overall reduction in complications for group of patients [11].

Globally, the use of HBPM by patients is gaining popularity. The prevalence of use ranges from 24% to 66% [13,14]. In the Asia Pacific region, a study in a district polyclinic in Singapore showed a prevalence of use of 24% [14]. Whereas, in Italy, a 2004 survey of hospital outpatient attendees found that 66% of them regularly use HBPM [13].

Patients with hypertension found it acceptable to monitor their blood pressure at home [15]. Primary care patients who were given home monitoring equipment by doctors to use at home reported no technical difficulty using the machine, and many welcomed the opportunity to be more involved in the management of their hypertension [16]. In this study, the patients that reported positive experiences with HBPM were observed to be ready for more involvement in the management of their hypertension. However, studies have also reported that many patients have purchased and used HBPM, even without the advice or guidance of their doctors [13,16].

The effect of purchasing and using HBPM at patients' own initiative has not been studied before. This study aimed to explore the practices and experiences of these patients. Using the qualitative approach, the study hoped to understand the influences of HBPM on these patients. The findings of this study will be useful for healthcare providers when faced with patients who self-initiated the use of HBPM as part of their hypertension management.

## Methods

The study was carried out at an urban primary care clinic, located within the University Malaya Medical Centre in Petaling Jaya, Selangor. Petaling Jaya covers an area of 51.4 km^2^, with an estimated population of a little over 450,000 inhabitants (2003 figures). The clinic served patients from all walks of life, ranging from the affluent middle class to patients from a lower socioeconomic status. As it is attached to a tertiary referral hospital, the clinic also sees patients from outside the district and state. Ethical approval for the study was obtained from the UMMC Ethics Committee on 22 April 2009, as per letter dated 24 April 2009 with MEC Ref. No: 715.2.

### Study Population

The populations sampled were primary care patients diagnosed with hypertension, who had experience owning and using home blood pressure monitors as part of their hypertension management. Purposive sampling methods were used to recruit patients who self-initiated the use of HBPM. Patients were informed of the study by their healthcare providers and through posters. Those who were interested gave their contact numbers to a member of the research team. Patients were then invited to attend either an in-depth interview session or a focus group discussion, based on their availability. Informed consent and simple demographic data including age, gender, who initiated the purchase and duration of HBPM usage were obtained prior to each data collection session.

Table 1 shows the characteristics of the 24 patients involved in the study. The sample size was determined by saturation of data where no new themes concerning the studied subject emerged.

**Table 1:** Characteristics of study participants

	In-depth Interview (n = 6)	Focus Group Discussions (n = 18)
**Age** **mean in years **(range)	64 (53-71)	60 (30-75)

**Ethnicity**		
**Malay**	2	6
**Chinese**	2	9
**Indian**	2	3

**Duration of HBPM use mean in years **(range)	9 (2-18)	5 (0.6-20)

**Years of Hypertension** mean in years (range)	12 (3-30)	10 (0.7-26)

**Occupations**	Retired clerk, teacher, housewife, retired officer, technical assistant, retired teacher	Retired clerk, teacher, housewife, retired officer, technical assistant, retired teacher, lorry driver

### Data Collection Methods

Patients' accounts with regards to HBPM were captured by two methods of data collection: in-depth interview and focus group discussions.

#### In-depth interviews

Six in-depth interviews were conducted to explore patients' practices and beliefs using HBPM as part of their hypertension management. A rough topic guide was used to act as a reminder of areas to be covered in the interviews (Additional file 1: Appendix 1). Interviews were conducted in both English and Bahasa Malaysia by the primary investigator and lasted for 40 -90 minutes.

#### Focus Groups

Two focus group discussions were undertaken involving eighteen patients. The patients in each focus group consisted of an approximately equal distribution of male to female patients, and all major ethnic groups were represented. The primary investigator (AA) acted as the moderator, and there was a research assistant who took notes from the discussions. The patients were asked general questions regarding their experience in using HBPM, and intra-group discussions were encouraged. The discussions were conducted in both English and Bahasa Malaysia. All interviews were digitally recorded, and field notes were also taken.

### Data Analysis

The data from the interviews and focus groups were transcribed verbatim. Data was analysed by the primary investigator (AA) using thematic analysis. The transcriptions were large, and had to be stored and managed accordingly whilst simultaneously ensuring confidentiality. Thus the qualitative software, NVivo 8 program was used for this purpose, as well as to assist in the handling of massive data and streamlining of the coding process [17]. The transcripts were read and re-read many times to "discover" or label variables as categories and their interrelationships. A second investigator (SO) also coded three of the interviews independently. Both investigators (AA, SO) then held discussions where the codes were compared, and any disagreements consolidated. After the initial stage of coding, 27 categories emerged from the data. The dismantled data was then brought together by axial coding process, under wider themes that connected the categories [18]. These themes are the ones that appear as the major findings. Emerging themes became a guide for further data collection. This also allowed for further enquiries into practices that were ambiguous or contradicting [17]. The data collection ceased once data saturation had been reached.

## Results

This study identified several influences of HBPM on patients' management of hypertension. These influences could be divided into positive and negative influences, depending on their potential effect on blood pressure control. Those that could improve blood pressure control were placed under positive influences, and those that could lead to worsening of blood pressure control placed under negative influences. From analysis of patients' responses, this study was able to identify five themes under positive influences and two themes under negative influences.

### A. Positive Influences of HBPM

#### 1. Increased Reflection of Their Lifestyle

Patients universally believed that their diet and exercise regime have an influence on their blood pressure readings. The readings they obtained from HBPM were frequently used as a form of feedback, to gauge the adequacy of their lifestyle changes. Patients reported altering their diet and increasing their frequency of exercise in response to high home blood pressure readings.

What do I do? I will note down what food I eat and what activity I did. That was during the holidays. I will note down today what did I eat, then I said maybe this food is good for me then I will note down whether I go for a walk in the morning, whether I sweat. I noticed when I sweat the pressure goes better

(IDI2/53-year-old/10 years of HBPM usage)

*When its high, then I know it's either my diet or I've not been exercising. So when it's high I'll take my walks. I'll do my aerobics and then cut down on meats, go vegetarian*.

(FGD2/68-year-old/3 years of HBPM usage)

#### 2. Increased Awareness and Interest in Hypertension Management

Patients in this study seemed knowledgeable about their hypertension treatment. Patients comfortably used medication names and dosages when discussing their medications. By using HBPM, they were able to see the effect of pharmacological treatment, and as a result were able to comment on the efficacy of different hypertension medications.

*My pressure is still high, so I wonder whether the pressure pills (is not effective). Because I was on Atenolol then changed to Coversyl. Now (I am) on Cozaar*.

(FGD2/63-year-old/10 years of HBPM usage)

It is better (with HBPM) because you can see whether the medicine is effective. Even now when I am on medicine already, before taking the medicine I will go and measure, after taking the medicine I will also measure. That's why I notice that Coversyl is more effective for me

(IDI2/53-year-old/10 years of HBPM usage)

This awareness and interest resulted in patients reporting improved adherence to their antihypertensive medications.

What causes the change in my blood pressure? I will relax and will observe the readings. I realized that I do need to take the medications because if I don't, the blood pressure will go up

(FGD1/54-year-old/1 year of HBPM usage)

#### 3. Sense of Reassurance

Patients felt reassured with their ability to continue monitoring their blood pressure at home. Many felt that the without it, duration until their next clinic follow-up appointment was too long, regardless of the actual duration. Home monitoring gave them a sense of confidence that their blood pressure readings were satisfactory between appointments.

Why we need the machine in the house? I think it's an important thing to have if we can afford because our visit to the doctor (is infrequent). Duration between visits is about three months

(FGD2/68-year-old/3 years of HBPM usage)

The doctor gave me 8 months appointment; I might have died by then

(FGD1/53-year-old/5 years of HBPM usage)

I can say this machine can give patient immediate psychological relaxation. It is very important. If you are not feeling well and the blood pressure reading is still normal. So feeling psychologically relax

(FGD1/66-year-old/20 years of HBPM usage)

Eighteen patients also obtained home blood pressure readings for reassurance when they experienced symptoms. These symptoms varied between patients, but they felt relieved with the ability to measure their blood pressure readings when symptoms occurred.

So usually it's like that (checking irregularly). Except when I feel very bad, like something is wrong. Sometimes I may feel some chest pains then I'll go and check

(FGD1/40-year-old/3 years of HBPM usage)

Aiyo, the days when I get the reading one two zero (120 mmHg), ahh so happy. That's like striking the first prize in lottery you know

(FGD2/63-year-old/7 years of HBPM usage)

#### 4. Doctor-Patient Therapeutic Alliances

The patients reported on-going discussions regarding their hypertension management with their healthcare providers, especially with regards to the home blood pressure readings. The processes had strengthened the therapeutic alliance, and increased trust between patient and healthcare providers.

The doctor told me to take the readings early morning after waking up from sleep, then at noon after being active and before bed time (all in all 3 readings). Then the readings I brought to show to the doctor

(FGD1/54-year-old/1 year of HBPM usage)

One patient noted his readings at home to be lower than the ones recorded in the clinic by his cardiologist. So he was asked by his healthcare providers to bring his home monitors to clinic for validation of accuracy.

I said that the doctor's manual blood pressure readings are incorrect. When I argued with him, he said bring your machine and check it against his manual machine

(FGD2/71-year-old/10 years of HBPM usage)

Many patients wanted to be told about blood pressure readings that are recorded in the clinic, and wanted more time with their doctors to discuss their home blood pressure readings and subsequent management.

But when we went for check-up, the doctor rarely tell us the actual blood pressure reading. He did not tell us the readings he just said it's okay. Actually we would like to know, the actual reading of the diastolic blood pressure

(FGD1/63-year-old/3 years of HBPM usage)

#### 5. Extended Use to Other Family Members

Some patients related that they had shared the use of home blood pressure machine with their spouse, parents and siblings. This led to enhanced awareness and support from their family members.

I have the machine in the house since 1998, because my husband is a diabetic. He and my mother both have diabetes and high blood pressure, so we decided to buy one (home blood pressure monitor). So when I check their blood pressures, I will also check mine

(FGD2/57-year-old/5 years of HBPM usage)

Definitely useful, that's why I taught my mum to use the meter. My meter is very easy to use, so she had been using it diligently

(IDI1/53-year-old/10 years of HBPM usage)

### B. Negative Influences of HBPM

#### 1. Unanswered Concerns and Confusion

Many patients expressed concern regarding the reliability of the home blood pressure readings and ultimately the accuracy of the digital blood pressure monitors. They noted variability in the blood pressure readings, and therefore showed a tendency to regard or disregard the values according to their own judgements.

That's why. I am also very suspicious, I mean, (the readings are) so variable within two weeks you know. Up and down, never consistent. So, we wonder whether I should be taking tablets. Is it worth of taking tablets or is it worth of using the machine at all, you see

(FGD2/75-year-old/10 years of HBPM usage)

Its measurement fluctuates, every moment of the day. (In the) morning will be 140 (and) later 120. Then I said why it is such a variation?

(FGD2/67-year-old/9 months of HBPM usage)

One of the younger patients tried to clear his confusion by discussing his home blood pressure readings with his doctor. He mentioned that this variation could be minimised by increasing the number of measurements taken at one time. This technique was taught to him by his healthcare provider.

Take two readings and repeat again. It could be quite accurate la, quite accurate. Take the average

(FGD2/30-year-old/5 years of HBPM usage)

Confusion also arose regarding the acceptable target blood pressure levels and the frequency of monitoring. One participant had uncontrolled hypertension for many years and had recently suffered from stroke. He believed that his target blood pressure must be compared against his previous blood pressure reading, and target value should be individualised.

Because my previous readings (systolic blood pressure (SBP)) are 179, 180(mmHg) and the lower reading 110 (mmHg). So when I get reading of (SBP) 159 (mmHg) and occasionally the lower reading (diastolic blood pressure) 90 (mmHg), I felt happy. So for me when I occasionally get the reading (SBP) 140 (mmHg), that is already very good for me

(FGD1/54-year-old/1 year of HBPM usage)

The frequency of checking blood pressure also varied. Some measured their blood pressure daily without fail. Others did it only when they had symptoms or when it was convenient. None had written the readings down in a record book.

I checked mine every day, without miss. One time before going to sleep

(FGD1/66-year-old/3 years of HBPM usage)

#### 2. Patients Making Own Treatment Decisions (Self- doctoring)

Some patients made their own decision as to whether to comply with medical advice based on the home blood pressure readings. These included starting or stopping of medications, as well as advice on lifestyle changes.

Based on home blood pressure readings, two patients delayed in taking hypertension medications although prescribed by their healthcare providers. One patient had diabetes, and her office blood pressure was off target (> 130/80 mmHg), but because her home blood pressure readings were around 125/80 mmHg, she decided not to take her medications.

But if you check, normally it doesn't go more than one twenty five on my machine. So when I come here they say it's high. That's why I begin to (doubt). But I never took the medication (given) but I exercise

(FGD2/57-year-old/5 years of HBPM usage)

Another patient said she was rushing when she arrived at the clinic room, which could explain the high office reading. When she rechecked the blood pressure reading at home, she felt this was acceptable and therefore she didn't take the medications added by her doctor for her hypertension.

Yesterday morning I checked (my BP) was alright. When I came (to the hospital) was higher the systolic. Then the evening I didn't started the medication yesterday. Last night I checked twice again, the readings were 150 over and 83 something. Of course I didn't start the medication

(FGD1/70-year-old/10 years of HBPM usage)

The variable blood pressure readings at home at times hindered some patients' adherence to medical advice. One patient had been struggling to come to terms with his diagnosis of hypertension. He noted that his blood pressure readings at home seemed to vary widely and did not change much despite medication. He began to question the need to take the medication.

But I have to take the same type (of antihypertensive medication). I checked my reading..... not different from what it was. Then should I stop?

(FGD2/75 years old/using for 10 years)

## Discussion

### Summary of Main Findings

This study assessed the influence of HBPM on hypertensive patients who self-initiated its use. In-depth exploration of patients' experiences and practices showed that self-initiation of HBPM is associated with both positive and negative implications.

### Positive Influences

The positive influences included being more reflective, especially concerning lifestyle changes, having an increased interest in the management of their hypertension and giving users feelings of reassurance. All these positive influences lead to improved patient's self-efficacy. Self-efficacy is defined as a person's confidence to carry out behaviour necessary to reach a desired goal [19]. It is an important precondition for successful self-management and behaviour change in patients with chronic disease such as hypertension. In this study, interviewed patients reported an increased adherence to pharmacological and non-pharmacological method of hypertension management when they noted high blood pressure readings on their home blood pressure monitors. A recent study on the use of HBPM in Japan reported that patients who used HBPM regularly are more likely to have satisfactory compliance with exercise diet and medications [20].

This study found that dealing with a disease that is mostly asymptomatic raised a great deal of anxiety and uncertainty on the part of the patients. The tangible readings from home blood pressure monitors turned this largely asymptomatic disease onto a condition that is measurable in patients' own homes. These readings became the patients' cue for action to take the necessary actions. Furthermore, patients in this study reported first hand observations of the effect of unhealthy practices such as a salty diet and sedentary lifestyle on their blood pressure readings. The HBPM readings reinforced advice given by healthcare practitioners on the appropriate lifestyle changes and the need for compliance.

This study also found that patients who self-monitor were eager to be more involved in discussions about their blood pressure control, especially when there is discrepancy between home and clinic blood pressure readings. Similar findings were also reported in a qualitative study looking at primary care patients' experiences of home blood pressure measurement in the United Kingdom [16]. When participants mentioned discussing the home blood pressure readings with their doctors, it was either to confirm the findings, or in an effort to delay or reduce the number of anti-hypertensive medications they are taking. Either way, the act of monitoring blood pressure at home suggested increase interactions between the patients and their doctors. It was found that the patients' blood pressure control could be improved within increased participation [21].

The importance of HBPM as a mode of hypertension screening in the community was revealed in this study. Patients reported sharing the use of HBPM with their immediate family, and occasionally even neighbours. In fact, some of the patients involved in this study reported using HBPM prior to being diagnosed with hypertension. With wider use of HBP monitors in the community, potentially more people with hypertension could be diagnosed. This is provided that patients are given greater knowledge regarding HBPM readings that could indicate diagnosis of hypertension and the HBP monitors used are accurate and validated.

### Negative Influences

Despite the benefits of HBPM, this study also found some negative influences of self-initiated HBPM on these patients. Patients reported feeling confused and unsure of the true meaning of HBP readings. They noted the values seemed to be fluctuating, and sometimes differed significantly from clinic readings. Since these patients self-initiated the purchase and use of HBPM, the machine purchased might not have been validated, and thus may not be completely accurate. In Turkey, only 40.7% of the HBP monitors used by patients were accurate, when checked in a campaign run at a University Hospital [22].

Only some of the patients in this study reported telling their doctors about their HBPM. Lack of time and doctor's lack of interest were some of the reasons given for not consulting their doctors. For those lacking guidance, the variability of HBPM readings led them to regard and disregard HBPM readings according to their own standard. The lack of guidance seemed to stem not only from lack of awareness, as some patients reported telling their doctors about their HBPM. Low level of acceptance by doctors could be another reason. A study in Canada noted that only 19% of the primary physicians surveyed used the home readings to guide therapy [23]. These doctors stated concerns about patients becoming preoccupied with measuring their blood pressure and the accuracy of home devices as the reasons for not fully utilising HBPM. It has also been showed that when reported by patients, the HBPM readings are seldom documented by doctors, thus limiting their use in future consultations [24].

### Implications for Further Research and Clinical Practice

From the findings in this study, primary care providers need to increase their participation, and give guidance to patients who self-initiated owning and using HBPM. As patients in this study reported using HBPM readings to decide on various aspects of their hypertension management, most critically whether to start taking new medication started by their doctors, doctors managing these patients need to discuss the significance of the HBPM readings and the target HBP reading which is different from the clinic reading. Other areas to cover in counselling sessions are information regarding validated brand names and models, correct techniques of measurement and actions to take when high readings are experienced. It is also important to be aware that people other than the patients may ultimately use the monitors. Advice on how to tackle these situations should also be given to patients.

The aspect of self-doctoring in patients using HBPM also needs to be regulated. It is impossible to eliminate all aspects of patient self management of their disease using home monitoring. From this study it is important to lay down ground rules concerning self-doctoring, such as discussions on the limitations of the home blood pressure readings, the importance of long term control and not just one off reading, and the concept of a risk continuum, where the aim is to reduce the overall cardiovascular risk and not just the blood pressure numbers. It is through the concerted effort of doctors and patients that the true potential of HBPM could be realised and its negative implications minimised.

### Limitations

This study is not without limitations. Although this study had a fair representation of primary care patients with hypertension, selecting the study population from a university-based urban primary care clinic limits the findings. Many of the patients are from the middle to high-income group, as well as coming from a more educated background. The findings may be more representative of more knowledgeable and interested patients. The voluntary nature of recruitment also may have led to over-representation of hypertensive patients who have greater motivation. This may lead to the increased interest in the involvement of hypertension management in this group of patients.

## Conclusions

HBPM is becoming an integral part of hypertension management. Primary care patients who self-initiated HBPM use need their doctors' participation and guidance especially regarding target blood pressure level, frequency of monitoring and measures to deal with aberrant blood pressure readings. Without which, the true benefits of this important self-management tool may not be realized.

## Competing interests

The authors declare that they have no competing interests.

## Authors' contributions

AA designed the study, conducted the interviews, analysed the data and drafted the manuscript. SO conducted one focus group discussion, analysed the data and provided overall supervision of the study. All authors were responsible with redrafting the final version of the manuscript. All authors read and approved the final manuscript.

## Appendix 1

### Topic Guide: In-Depth Interview and Focus Group Discussion

1. The purchase

a. Impetus

b. Relationship with disease

c. Encouragement/triggering event

d. Discussion/encouragement from doctor

2. The choice

a. Factors in consideration: price, accuracy, validation

b. Use of other health related monitors

3. The usage

a. Intended use

b. Actual use

c. Explanation of technique

d. Frequency of use/influencing factors

4. The readings

a. What is considered normal?

b. What do they do with the readings?

c. How did the readings influence the management of hypertension?

d. What do they do if the readings deemed too high/low?

e. Any discussion of the readings with the doctor?

5. The value

a. Perceived value

b. Actual value

c. Any barrier to maximize the value

d. Any potential harm

6. The difficulties

a. Any difficulties with use

b. How?

c. Calibration/durability issues

## Pre-publication history

The pre-publication history for this paper can be accessed here:

http://www.biomedcentral.com/1471-2296/12/143/prepub

## Supplementary Material

Additional file 1 **Appendix 1: Topic Guide: In-Depth Interview and Focus Group Discussion**. Click here for file
