# Supplementary material for: The influence of self-owned home blood pressure monitoring (HBPM) on primary care patients with hypertension: A qualitative study
Source: BMC Fam Pract. 2011 Dec 30;12:143. doi: 10.1186/1471-2296-12-143 (PMC3271963; doi:10.1186/1471-2296-12-143)
Supplement: Additional file 1 — Appendix 1: Topic Guide: In-Depth Interview and Focus Group Discussion. [file 1471-2296-12-143-S1.PDF]

Appendix 1

TOPIC GUIDE: IN-DEPTH INTERVIEW AND FOCUS GROUP DISCUSSION

1. The purchase
    a. Impetus
    b. Relationship with disease
    c. Encouragement/triggering event
    d. Discussion/encouragement from doctor
2. The choice
    a. Factors in consideration: price,accuracy,validation
    b. Use of other health related monitors
3. The usage
    a. Intended use
    b. Actual use
    c. Explanation of technique
    d. Frequency of use/influencing factors
4. The readings
    a. What is considered normal?
    b. What do they do with the readings?
    c. How did the readings influence the management of hypertension?
    d. What do they do if the readings deemed too high/low?
    e. Any discussion of the readings with the doctor?
5. The value
    a. Perceived value
    b. Actual value
    c. Any barrier to maximize the value
    d. Any potential harm
6. The difficulties
    a. Any difficulties with use
    b. How?
    c. Calibration/durability issues
